# Maternal exposure to genistein during pregnancy and oxidative DNA damage in testes of male mouse offspring

**DOI:** 10.3389/fnut.2022.904368

**Published:** 2022-07-18

**Authors:** Roger W. L. Godschalk, Margit C. M. Janssen, Kimberly Vanhees, Sahar Barjesteh van Waalwijk van Doorn-Khosrovani, Frederik-Jan van Schooten

**Affiliations:** Department of Pharmacology & Toxicology, School for Nutrition and Translational Research in Metabolism (NUTRIM), Maastricht University, Maastricht, Netherlands

**Keywords:** genistein, estrogen metabolism, testes, antioxidant system, DNA damage

## Abstract

**Background:**

Genistein is a dietary supplement with phyto-estrogenic properties. Therefore, high intake of genistein during pregnancy may have adverse effects on the genetic integrity of testes and germ cells of male offspring. In this study, we examined whether maternal exposure to genistein during pregnancy induced oxidative DNA damage in the male germline at adolescence.

**Methods:**

*Atm*-ΔSRI mice have lower glucose-6-phosphate dehydrogenase (G6PDH) activity, which is important for maintaining levels of reduced glutathione and therefore these mice have an increased susceptibility to oxidative stress. Parental heterozygous *Atm*-ΔSRI mice received a genistein-rich or control diet, after which they were mated to obtain offspring. During pregnancy, mothers remained on the respective diets and after delivery all animals received control diets. Redox status and oxidative DNA damage were assessed in testes and sperm of 12 weeks old male offspring. Gene expression of *Cyp1b1, Comt*, and *Nqo1* was assessed in testes, and DNA methylation as possible mechanism for transmission of effects to later life.

**Results:**

Intake of genistein during pregnancy increased oxidative DNA damage in testes of offspring, especially in heterozygous *Atm*-ΔSRI mice. These increased DNA damage levels coincided with decreased expression of *Comt* and *Nqo1*. Heterozygous *Atm*-ΔSRI mice had higher levels of DNA strand breaks in sperm compared to wild type littermates, and DNA damage was further enhanced by a genistein-rich maternal diet. G6PDH activity was higher in mice with high maternal intake of genistein compared to control diets, suggesting compensation against oxidative stress. A positive correlation was observed between the levels of DNA methylation and oxidative DNA damage in testes.

**Conclusion:**

These data indicate that prenatal exposure to genistein altered gene expression and increased DNA damage in testes and sperm of adolescent male offspring. These effects of genistein on DNA damage in later life coincided with alterations in DNA methylation.

## Introduction

Phyto-estrogens are plant-derived compounds that resemble estrogens in their molecular structure and size, in particular the primary female sex hormone 17β-estradiol (E2). Because of their structural similarity to E2, phytoestrogens are able to bind to the estrogen receptor (ER) and subsequently act as estrogen agonists or antagonists that may either enhance or block the downstream effects of the ER (1, 2). Isoflavones are a group of phytoestrogens that show the greatest estrogenic effect and their main food source is soy and soy-derived products. However, they are also present in other plant products including chickpeas, beans, fruits, nuts, and vegetables (3). Due to their ability to act on ERs, isoflavones contain endocrine-disrupting properties. It is important to explore these biological effects especially during pregnancy, as isoflavones are able to cross the placenta and reach the umbilical cord blood, amniotic fluid, and fetal tissues (4, 5). Besides their ability to bind to ERs, isoflavones exert antioxidant activity (6) by decreasing the levels of reactive oxygen species and by increasing the expression of antioxidant enzymes. By virtue of these antioxidant properties (7), plant-based therapies or dietary supplementation with high doses of phyto-estrogens have been recommended.

Genistein is a common ingredient of dietary supplements. It is an isoflavone that contains a diphenol structure and a similar distance between the hydroxyl groups as E2, enabling genistein to bind to the ERα and ERβ subtypes (8). Through both ER-dependent and -independent signaling, genistein is suggested to have a beneficial role in the modulation of oxidative stress and inflammation, prevention of cancer and diabetes, and reduction of post-menopausal symptoms (9–11). In contrast to these suggested positive effects of genistein on health, the endocrine-disrupting properties of genistein is a matter of concern. Examples of the role of genistein in reproductive and developmental disorders are the associations between increased genistein exposure and altered hormone levels and suppression ovulation (12, 13).

**Figure 1:**
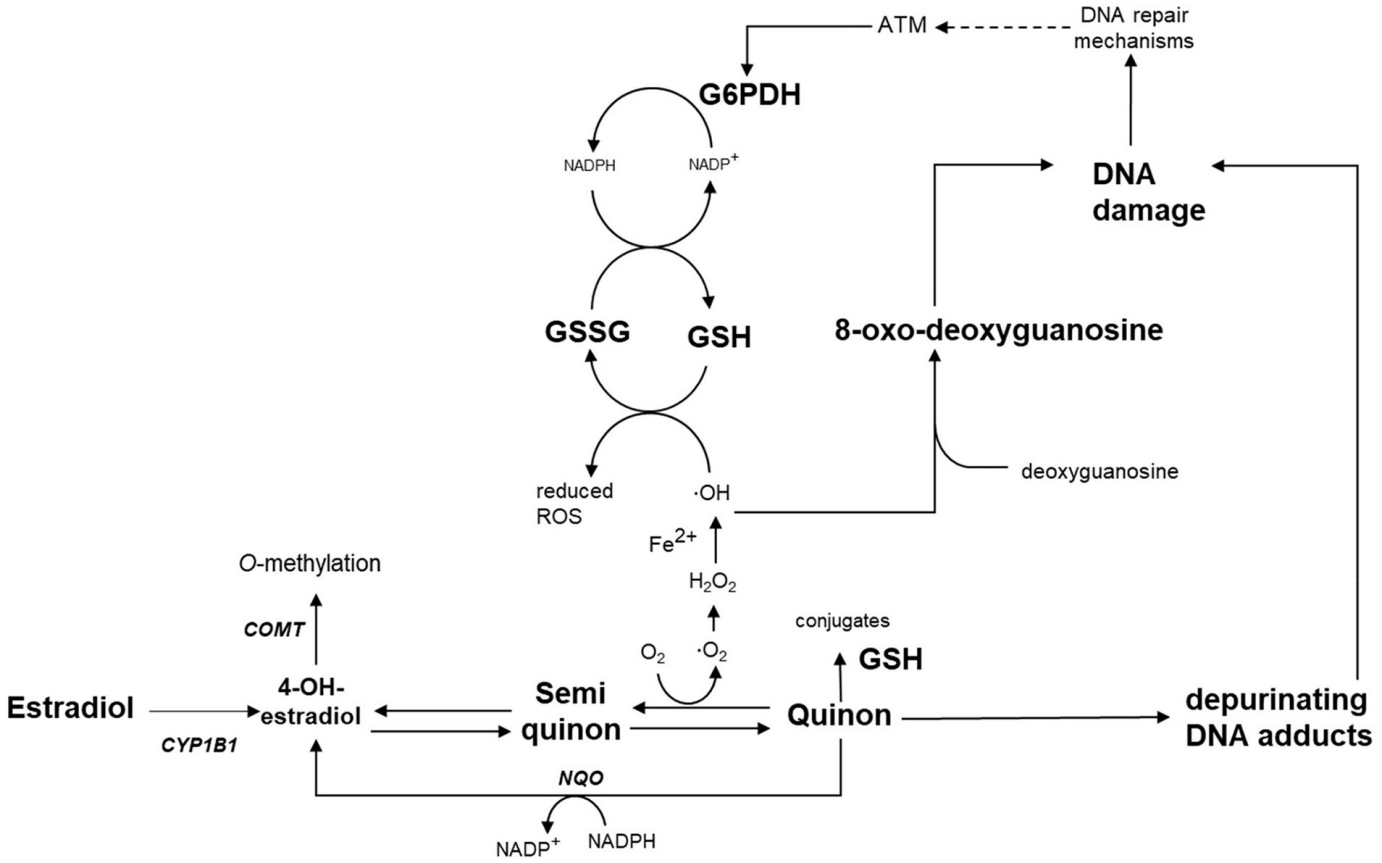
Schematic overview of the hypothesis of this study: Estradiol is metabolized by Cyp1b1 to catechol estrogens (e.g., 4-OH-estradiol) and catechol estrogen-quinones, which undergo redox cycling leading to oxidative stress. Redox cycling can be prevented by detoxification *via* Comt, Nqo1 or conjugation with glutathione. Exposure to genistein during pregnancy is expected to permanently alter the expression of *Cyp1b1, Comt* and *Nqo1*, and can alter G6PDH activity. These alterations lead to oxidative stress and DNA damage in the male germ line later in life. COMT, catechol-*O*-methyltransferase; CYP1B1, cytochrome P450 1B1; G6PDH, glucose-6-phosphate dehydrogenase; GSH, glutathione; GSSG, glutathione disulfide; NQO, NADPH-quinone-oxidoreductase; ROS, reactive oxygen species.

As described by the concept of developmental programming, adverse environmental exposures during crucial *in utero* periods of development may have long-term effects on the structure or function of an organism (14, 15). Therefore, exposing the fetus to increased concentrations of genistein could cause permanent alterations in physiological responses, resulting in functional impairments and disorders in adult life, supposedly involving the expression of anti-oxidants and the metabolism of estrogens. The developmental programming concept predicts that the fetus adapts to the high intake of antioxidants by lowering its own anti-oxidant defenses. If genistein is no longer supplemented after birth, these alterations may lead to increased oxidative stress, because the own anti-oxidant system can no longer adapt sufficiently. A second proposed mechanism, would be that genistein acts as a phyto–estrogen during pregnancy and the fetus thus adapts its metabolism of estrogens. Alterations in the estrogen metabolism in male offspring seem unimportant, because estrogens are considered to be female hormones. However, the importance of estrogens in males is demonstrated by the failure of spermatogenesis in male mice with a deficiency in aromatase (16) and by infertility in mice that carry ERα gene knockouts (17). In addition to the increased susceptibility for developing functional impairments, genistein-induced alterations in estrogen metabolism could possibly lead to increased oxidative DNA stress in testes, due to increased redox cycling and formation of reactive oxygen species (ROS) (Figure 1) (18). As a result, DNA damage in the testes and male germ cells may be increased, which is a hazard for the genetic integrity of future generations. To prevent this damage, it is important to understand to what extent the endogenous antioxidant system is able to cope with the increased levels of ROS. To this end, the links between maternal genistein-intake, estrogen metabolism and the antioxidant system in testes of the offspring should be further investigated. Therefore, the aim of this study was to examine the effect of maternal genistein-intake during pregnancy on gene expression of genes involved in the metabolism of estrogens (*Cyp1b1, Comt*, and *Nqo1)*, the antioxidant capacity in testes of male offspring, and the subsequent genetic integrity in sperm. An *Atm*-ΔSRI animal model (19) was used to study the additional effect of having a compromised antioxidant and DNA repair system.

## Materials and methods

### Mice and sample collection

**Figure 2:**
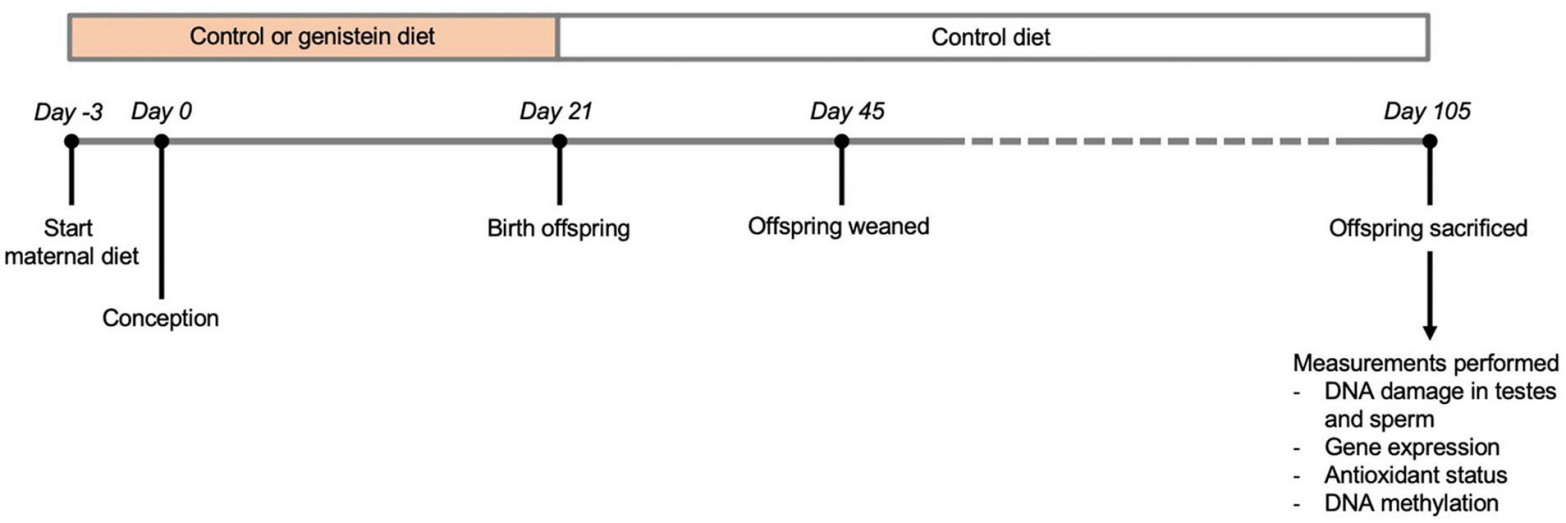
Overview of study design.

The breeding of mice and sample collection was performed as described previously (19). Briefly, the parental animals received at 8 weeks of age either normal (control) chow (*n* = 8; complete feed for mouse breeding with low-phytoestrogen content lacking both soybean and alfalfa products, resulting in minimal levels of genistein, daidzein, and coumestrol; Ssniff, Soest, Germany) or chow supplemented with genistein (*n* = 9; 270 mg/kg feed; LC Laboratories, Woburn, MA, USA) from 3 days before conception until birth of offspring (Figure 2) (20). Male and female Heterozygous *Atm*-ΔSRI mice (129/SvJ: C57BL/6J background) were mated to obtain littermates of three *Atm* genotypes; wild type (Wt), heterozygous *Atm*-ΔSRI (Hz), and homozygous *Atm*-ΔSRI (Mut) mice. After birth, all mothers and offspring received the control diet, ensuring that the prenatal period was the only window of possible genistein exposure. Five days after birth (day 26), the weight, sex and *Atm*-ΔSRI genotype of the pups were determined using a standard polymerase chain reaction (PCR) procedure with DNA obtained from tail-cuts. Twelve weeks after birth (day 105), the pups were anesthetized and sacrificed by cardiac puncture. The epididymides and the testes were isolated, after which the epididymides were cut open and washed in PBS thereby isolating the sperm, and the testes were snap-frozen in liquid nitrogen. Of each genotype (Wt, Hz, and Mut), 9, 18, and 10 male mice offspring were used for analysis respectively, in which one sample represents the tissue from 1 male mouse and only one Wt, Hz or Mut mouse from each litter were used to prevent nest-effects. The mouse experiments were conducted in accordance with Dutch animal protection laws by the guidelines of the Institutional Animal Care Committee of Maastricht University.

### Oxidative DNA damage assessment in the testes by HPLC-ECD

DNA was isolated from homogenized testis in the presence of the anti-oxidant hydroxyquinoline to prevent artificial induction of oxidative DNA damage (21). DNA concentrations and purity were determined specrophotometrically at 230, 260, and 280 nm. Oxidative DNA damage in the testes was determined as described previously (21) and was defined as the ratio of 8-oxo-2′-deoxyguanosine (8-oxo-dG) to normal deoxyguanosine. In brief, DNA was digested into deoxyribonucleosides by alkaline phosphatase (0.014 U/μl) and nuclease P1 (0.02 U/μl), after which the digest was injected into an HPLC, with a Gynkotek 340 isocratic pump (Gynkotek, Bremen, Germany) connected to a Supercosil™ LC-18S column (250 ×4.6 mm) (Supelco Park, Bellefonte, PA) and an electrochemical detector (Antec, Leiden, Netherlands). The mobile phase contained 10% aqueous methanol, and 90% buffer consisting of 13 mM K_2_HPO_4_, 94 mM KH_2_PO_4_, 0.5 mM ethylenediaminetetraacetic acid, and 26 mM NaCl. A flow rate of 1.0 ml/min was used. A lower detection limit of 40 fmol was reached for 8-oxo-dG or 1.5 residues/10^6^ 2′-deoxyguanosine. At the same time, deoxyguanosine was monitored spectrophotometrically at 260 nm.

### DNA damage assessment in sperm by comet assay

Single strand breaks were assessed by the comet assay, because these may represent both DNA damage by the formation of ROS as well as the formation of depurinating DNA adducts of estrogens or estrogen-like compounds (see Figure 1). The protocol for the comet assay is based on the technique used by Singh et al. (22) and adapted for sperm analysis (23). Sperm samples were resuspended in low melting point agarose and put onto standard glass slides that were pre-coated with 1% normal melting point agarose. Cell lysis was performed through submerging the slides in two successive lysis solutions; lysis buffer 1 (2.5 M NaCl, 100 mM EDTA, 10 mM Trizma^®^ base, pH 10, 1% Triton X-100) containing 10 mM dithiothreitol (DTT) at 4°C for 60 min, and lysis buffer 2 consisting of 0.05 mg/ml proteinase K at 4°C for 60 min. The DNA was denatured by incubation in electrophoresis buffer (pH 13.2) at 4°C for 60 min. After incubation, the slides were subjected to electrophoresis at 25 V (0.8 V/cm over the platform, 300 mA) for 30 min at 4°C. The slides were neutralized (0.4 M Trizma^®^ base, pH 7.5) three times for 5 min, and stained with ethidium bromide. The final read-out was the percentage DNA in the tail using CometIII software (Perceptive Instruments).

### Quantitative real-time PCR and methylation sensitive McrBC real-time PCR in testes

Quantitative real-time PCR was performed as described previously (24). In short, testes of 12-week-old mice offspring mice were homogenized by using the Ultra-Turrax homogeniser (IKA, Staufen, Germany). RNA was isolated by using TRIzol Reagent (Invitrogen, Breda, Netherlands) following the manufacturer's instructions. 1 μg RNA was used to create complementary DNA (cDNA) by using iScript reverse transcriptase (Bio-Rad, Hercules, CA, USA) according to the manufacturer's instructions. For the quantitative PCR amplification, an aliquot of one-tenth of the cDNA was applied, together with 7.5 pmol of each primer (Supplementary Table 1; Eurogentec, Maastricht, Netherlands) and 12.5 μl SYBR Green (Qiagen, Venlo, Netherlands) in a volume of 25 μl. A MyiQ single color RT-PCR detection system (Bio-Rad) was used to carry out the reactions. The PCR cycling conditions included one cycle of 3 min at 95°C, 40 cycles of 15 s at 95°C and for 1 min at 60°C, and one cycle of 1 min at 95°C for 1 min at 65°C. Data analysis was performed by using the MyiQ Software system (Bio-Rad), in which *C*_t_ values were normalized for an endogenous reference gene (β-actin) compared with the calibrator (i.e., average *C*_t_ value of control samples). The *C*_t_ values were expressed as fold change (2^−Δ*ΔCt*^).

The methylation pattern of repetitive elements was analyzed by using the methylation-sensitive McrBC real-time PCR assay (25). McrBC is an endonuclease that cleaves DNA containing 5-methylcytosine and does not act upon unmethylated DNA, thereby preventing amplification of methylated DNA in the PCR assay. The McrBC real-time PCR assay was performed as described previously (20). 10 U of McrBC (New England Biolabs, Beverly, MA, USA) was used to digested 1 μg of genomic DNA overnight at 37°C. A 2-step quantitative real-time PCR was performed. IQ SYBR Green Supermix (Bio-Rad Laboratories, Veenendaal, Netherlands) was used, with 25 pmol of each primer (Supplementary Table 2; Eurogentec, Maastricht, Netherlands) and 4 ng of McrBC-digested DNA in a reaction volume of 25 μl. The following forward and reverse primer sequences for the different repetitive elements and endogenous reference were used: short interspersed nucleotide element B1 (SINEB1), SINEB2, long interspersed nucleotide element (LINE), intracisternal A-particle (IAP) GAG, major satellite, and minor satellite. The PCR cycling conditions included an initial denaturation for 10 min at 95°C, after which 40 cycles for 45 s at 95°C and 90 s at 58°C were completed. For the major and minor satellites, this step was executed at a temperature of 60°C, using the iCycler (Bio-Rad Laboratories). Data analysis was performed by a MyiQ software system (Bio-Rad Laboratories), in which *C*t values were normalized for endogenous reference (*HPRT*) compared with the calibrator (i.e., average *C*t value of 3 control samples for the fetuses and four control samples for the adult mice). The *C*_t_ values were expressed as relative expression compared to controls (2^−Δ*ΔCt*^).

### Trolox equivalent antioxidant capacity assessment in the testes

A total antioxidant capacity assay was performed as previously described (26) to analyze tissue homogenates of the testes. 950 μl of an 2,2-azinobis-(3-ethylbenzothiazoline-6-sulfonate) (ABTS) radical solution was incubated at 37°C for 1 min, followed by the addition of 50 μl of tissue homogenate. The solutions were incubated for 5 min. and the absorption was subsequently measured at 734 nm. After 5 min, the decrease in absorption relative to the blank (buffer) was related to the decrease of Trolox calibrators. The value for Trolox equivalent antioxidant capacity (TEAC) represents the concentration of Trolox that has a similar antioxidant capacity as the sample in μmol/g testis.

### GSH and GSSG concentration assessment in the testes

The assessment of the cellular redox status in the testes was performed by quantifying the glutathione (GSH) to glutathione disulfide (GSSG) ratio. According to the enzymatic recycling method that quantifies the conversion of 5,5'-dithio-bis (2-nitrobenzoic acid) (DTNB) into 5'-thio-2-nitrobenzoic acid (TNB) spectrophotometrically at 412 nm and 37°C, total GSH content and GSSG were measured. The GSH/GSSG ratio was calculated from the content of GSSG and total GSH (27).

### Glucose-6-phosphate dehydrogenase activity in the testes

Glucose-6-phosphate dehydrogenase (G6PDH) activity was measured by assessing the conversion of NADP^+^ to NADPH on a Cary 50 UV-vis spectrophotometer (Santa Clara, USA) at 340 nm, using tissue homogenates of the testes in the presence of glucose-6-phosphate (19). The final incubation mixture consisted of 10 mM of MgCl_2_, 0.7 mM NADP^+^ and 2 mM glucose-6-phosphate, and a Tris-HCl (75 mM) buffer, pH 74 held at 37°C. By adding the tissue extract containing the enzyme or positive and negative controls, the reaction was started. G6PDH activity was expressed as units/gram tissue.

### Statistical analysis

Data are presented as mean ± standard error of the mean (SEM). Differences between groups were analyzed by the non-parametric Kruskal Wallis tests and *post-hoc* Mann-Whitney tests. Linear regression analysis was performed to analyze the relationship between DNA methylation and 8-oxo-dG levels. *P* <0.05 was considered as statistically significant.

## Results

### Analysis of DNA damage in the testes and sperm

**Figure 3:**
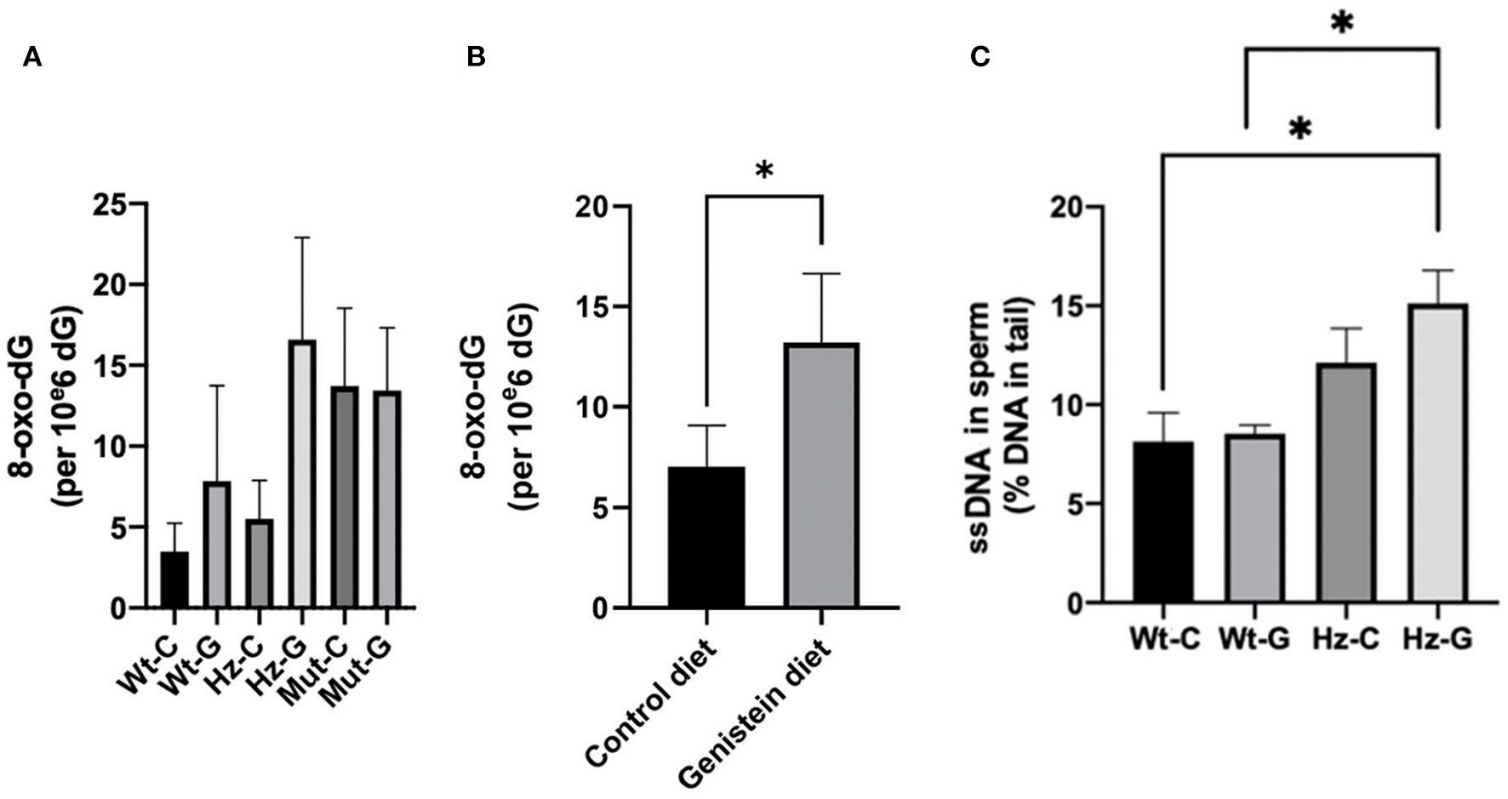
DNA damage in testes and sperm of mice with different maternal diets and various *Atm*-ΔSRI genotypes. Oxidative DNA damage determined by 8-oxo-dG levels in testes of **(A)** the various genotype-maternal diet combinations and **(B)** of groups with different maternal diets irrespective of their genotypes. **(C)** Single strand breaks in epididymal sperm presented as percentage DNA in the tail (*n* = 4 in each group). The difference between the wild-type control diet and the heterozygote-genistein diet group (*P* = 0.043), and the wild-type genistein diet and heterozygote-genistein diet group (*P* = 0.020) were statistically significant. C, maternal control diet; G, maternal genistein-rich diet; Wt, mice carrying the wild-type *Atm* gene; Hz, mice that were heterozygous for the *Atm*-ΔSRI mutation; Mut, mice that were homozygous for the *Atm*-ΔSRI mutation. Data presented as mean ± standard error of the mean (SEM). *P < 0.05.

Oxidative DNA damage in testes and sperm was assessed by 8-oxo-dG levels in the whole testes and DNA single strand breaks (ssDNAs) in sperm, respectively. No overall significant difference was found in 8-oxo-dG levels in the testes between the various genotype-maternal diet combinations (*P* = 0.085; Figure 3A). However, when the analysis was performed based on maternal diet only (i.e., pooling the animals with various *Atm*-ΔSRI genotypes), it appeared that maternal genistein diet significantly increased the level of 8-oxo-dG in the testes from 7.0 ± 2.1 (*n* = 18) to 13.2 ± 2.4 (*n* = 11) per 10^6^ dG (*P* = 0.047; Figure 3B). Maternal genistein diet did not increase DNA damage in the testes of homozygous *Atm*-ΔSRI mutant mice, because they already had “spontaneously” increased levels of 8-oxo-dG (see Figure 3A) (19). Additionally, male *Atm*-ΔSRI homozygous mice have no production of sperm. Therefore, the analysis of DNA damage in sperm by the comet assay was limited to wild-type and heterozygous mice (Figure 3C). The relative amount of ssDNA damage in the sperm was higher in mice that were heterozygous for the *Atm*-ΔSRI mutation, and a maternal genistein diet further enhanced this difference (*P* = 0.039), because no statistically significant difference was observed between control and Atm-ΔSRI animals that received a control diet during pregnancy.

Since homozygous *Atm*-ΔSRI mice already have spontaneously elevated levels of 8-oxo-dG in their testes and impaired sperm production, these mice were excluded from further analyses.

### Gene expression analysis

**Figure 4:**
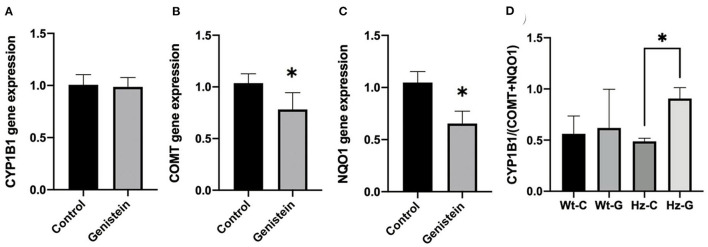
Gene expression changes in *Cyp1B1* **(A)**, *Comt* **(B)**, and *Nqo1* **(C)** between offspring animals according to maternal diet with (*n* = 9) or without genistein (*n* = 19), irrespective of the animals' genotype. **(D)** Ratio between gene expression of *Cyp1b1* and *Comt* plus *Nqo1*, representing the net effect of the changes in gene expression normalized to β-actin as house-keeping gene. C, maternal control diet; G, maternal genistein-rich diet; Wt, mice carrying the wild-type *Atm* gene; Hz, mice that were heterozygous for the *Atm*-ΔSRI mutation. Data is presented as mean ± standard error of the mean (SEM). *P < 0.05.

Gene expression of *Cyp1b1* in testes of male mice was not different between controls and genistein supplemented animals (Figure 4A). However, *Comt*, and *Nqo1* were significantly lower in mice with a maternal genistein-rich diet when compared to a maternal control diet (*Comt, P* = 0.02; *Nqo1, P* = 0.003) (Figures 4B,C). A ratio was calculated between the gene expression of enzymes that would contribute to the metabolism of estrogens toward metabolites that can undergo redox cycling and can form depurinating DNA adducts (i.e., *Cyp1b1)* and the sum of expression of enzymes that have a putative protective effect against redox cycling, ROS generation and DNA adducts (i.e., *Comt* and *Nqo1*). A significant increase in this ratio was observed in mice that were heterozygous for the *Atm*-ΔSRI mutation with a maternal genistein-rich diet (0.91 ± 0.03), compared to mice with a maternal control diet (0.49 ± 0.11 *P* = 0.002) (Figure 4D). This significantly higher ratio was predominantly due to decreased expression in *Nqo1*, and indicated that a maternal genistein-rich diet compared to control diet in heterozygous *Atm*-ΔSRI mice alters gene expression in favor of the development of ROS and depurinating DNA adducts (also see Figure 1). We realize that gene-expression does not necessarily reflect actual enzyme activity, but unfortunately, we had insufficient amount of material available to include enzyme activity measurements.

### Analysis of the antioxidant system in the testes

**Figure 5:**
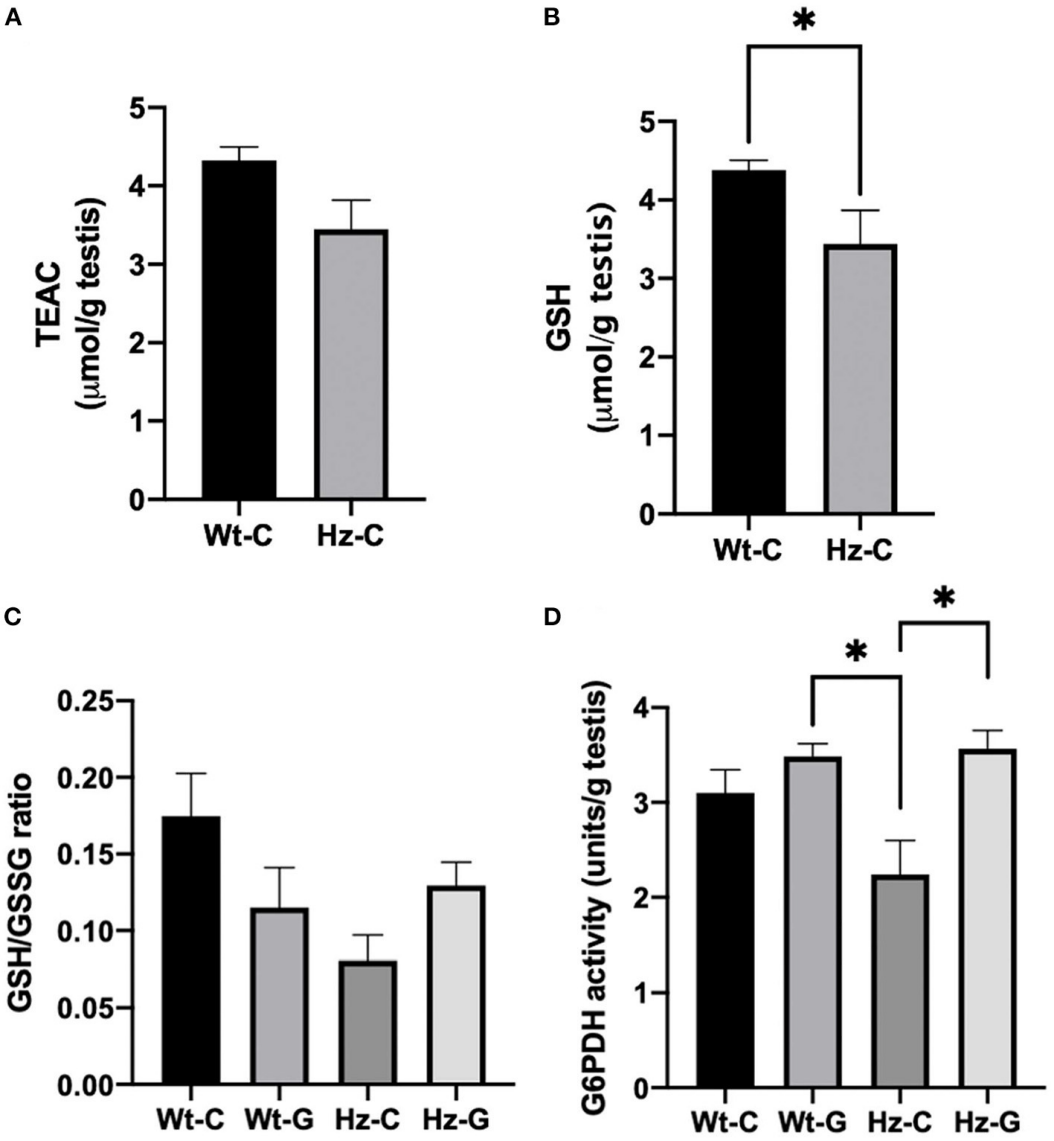
Redox status of testes of mice exposed to different maternal diets during pregnancy and various *Atm*-ΔSRI genotypes. **(A)** Trolox equivalent antioxidant capacity (TEAC) in μmol/g testes (*n* = 8 in each group). **(B)** Concentration of reduced GSH in μmol/g testes (*n* = 8 per group). **(C)** The ratio between reduced GSH and GSSG (*n* = 4 per group) and **(D)** G6PDH activity in units/g testes as assessed by measuring the conversion of NADP+ to NADPH in the same animals. C, maternal control diet; G, maternal genistein-rich diet; Wt, mice carrying the wild-type *Atm* gene; Hz, mice that were heterozygous for the *Atm*-ΔSRI mutation. Data presented as mean ± standard error of the mean (SEM). *P < 0.05.

We previously described that the Atm-ΔSRI mutation decreases the production of GSH (19). Indeed, both the TEAC as well as the concentration of GSH were lower in mice with a maternal control diet that were heterozygous for the *Atm*-ΔSRI mutation compared to mice with a wild-type genotype (Figures 5A,B). However, only the difference in GSH reached statistical significance (*P* = 0.043) with 3.4 ± 0.4 μmol/g in heterozygotes and 4.4 ± 0.1 μmol/g in wild-type animals. A positive correlation was found between the TEAC and the GSH concentrations (*R*^2^ = 0.887, *P* < 0.000). The GSH/GSSG ratio was lower in mice that were heterozygous for the *Atm*-ΔSRI mutation compared to the wild-type mice (Figure 5C). The effect of a maternal genistein-rich diet was different for each genotype group, as the GSH/GSSG ratio was lower in wild-type mice with a maternal genistein-rich diet compared to a control diet, whereas the GSH/GSSG ratio was higher in heterozygous mice with a maternal genistein-rich diet compared to the maternal control diet. However, these differences in GSH/GSSG ratio were non-significant (*P* = 0.069). This increase in GSH/GSSG ratio in testis of Hz animals may be the result of a compensation by increasing G6PDH activity. The G6PDH activity in the mice that were heterozygous for the *Atm*-ΔSRI mutation and had a maternal genistein enriched diet (3.564 ± 0.196 units/g) was significantly higher compared to the heterozygous mice with a maternal control diet (2.2 ± 0.4 units/g, *P* = 0.034) (Figure 5D).

### DNA methylation analysis in the testes

**Figure 6:**
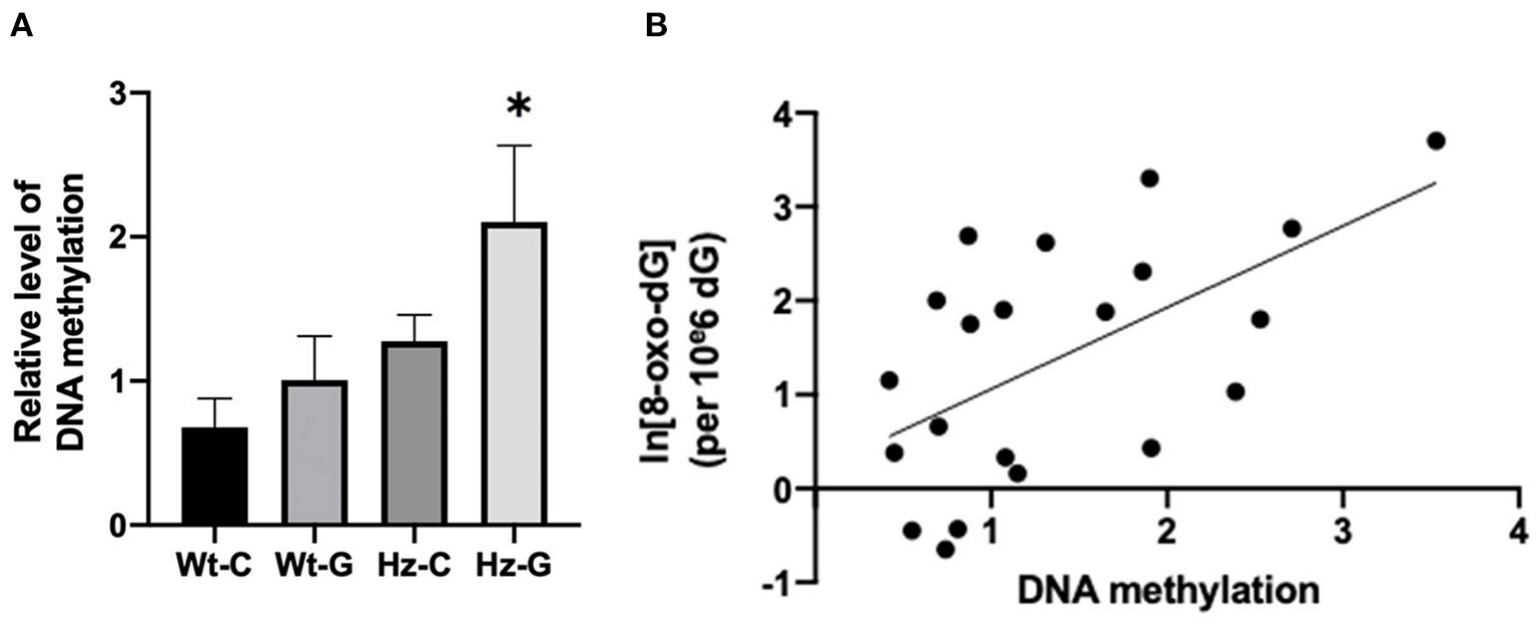
DNA methylation levels of mice with different maternal diets and *Atm*-ΔSRI genotypes and the correlation with oxidative DNA damage. **(A)** Relative levels DNA methylation measured by methylation sensitive McrBC real-time PCR assay using Hprt as reference gene. **(B)** A linear relationship between DNA methylation and the logarithm of 8-oxo-dG levels. C, maternal control diet; G, maternal genistein-rich diet; Wt, mice carrying the wild-type *Atm* gene; Hz, mice that were heterozygous for the *Atm*-ΔSRI genotype. Data presented as mean ± standard error of the mean (SEM). *P < 0.05.

Long lasting effects of maternal diets can be transmitted to later in life by changes in DNA methylation patterns. Therefore, the possible effect of maternal diet on global DNA methylation in mouse testes was examined. Animals that received a maternal genistein diet and had the heterozygous *Atm*-ΔSRI genotype appeared to have higher levels of DNA methylation compared to their Wt littermates (Figure 6A, *P* = 0.011). Furthermore, a positive correlation was found between the 8-oxo-dG levels and DNA methylation (*R*^2^ = 0.338, *P* = 0.006; Figure 6B).

## Discussion

The current study demonstrates that *in utero* exposure of male mice to a genistein-rich maternal diet results in significantly higher levels of DNA damage in testes when compared to mice with a maternal control diet, as measured by levels of 8-oxo-dG. Moreover, DNA strand breaks in sperm were also increased by maternal genistein intake, but only in mice with an antioxidant insufficiency due to an *Atm-*ΔSRI mutation. These findings suggest that a maternal genistein-rich diet may affect the genetic integrity of the germ line, which could thus have consequences for following generations. Although intake of genistein supplements during pregnancy is not recommended, young women of reproductive age may take these supplements even before knowing that they are pregnant. Over the counter genistein supplements are marketed to the general public, because of the numerous claims about health benefits. It is therefore important that the public is also informed about the potential risks associated with supplementation.

The higher levels of 8-oxo-G in the testes and single strand breaks in sperm of mice with a mutation in the *Atm*-ΔSRI gene, could be attributed to the function of the ATM serine/threonine kinase. ATM is often seen as a sensor or mediator in the regulation of the global cellular response to oxidative stress (28). It additionally controls the level of double strand breaks, which initiate meiotic recombination and the segregation of homologous chromosomes. Therefore, ATM is of great importance in proper spermatogenesis (29). Hence, lack of functional ATM resulted in “spontaneously” high levels of oxidative DNA damage and impaired spermatogenesis, as observed in mice homozygous for the *Atm*-ΔSRI mutation. The higher levels of oxidative DNA damage in sperm of the mice that were heterozygous for the *Atm*-ΔSRI mutation could therefore not only have been due to insufficient protection against ROS, but also because of insufficient DNA repair during meiosis (19). The effect of an *in utero* genistein-rich maternal diet on oxidative DNA damage in the testes could be explained by the hypothesized model (see Figure 1), in which genistein supplementation during pregnancy induced changes in estrogen metabolism that persisted into adolescence. These changes may result in the generation of ROS and the formation of estrogen metabolites that can form depurinating DNA adducts. Furthermore, we also analyzed pathways that might have prevented the harmful effects of estrogen redox cycling, including the expression of *Comt* and *Nqo1*. Gene expression of both *Comt* and *Nqo1* were indeed decreased after maternal genistein exposure, whereas expression of *Cyp1b1* was unaffected. Similarly, in an *in vitro* study by Wagner et al. (30), genistein inhibited gene expression of *COMT* and *NQO1*, and only slightly affected *CYP1B1*. These metabolic changes stimulated the formation of E2 genotoxic metabolites, and inhibited the detoxification of estrogen metabolites. However, it should be stressed that the study of Wagner et al. studied direct effects of genistein, whereas in our study the effect of prenatal dietary exposure was investigated. Since the mice in our study did not receive genistein enriched diets after birth, the effects of genistein on gene expression seem to be imprinted in the offspring's testes. One potential mechanism that could serve as cellular “memory” leading to differential gene expression is DNA methylation. For instance, a study by Wu et al. (31) showed that both E2 and genistein induced CpG site-specific methylation in the promotor region of *COMT*, thereby causing a decrease in *COMT* transcript levels. This finding is also in accordance with other studies in which genistein affected DNA methylation (32). By exposing the fetus to relatively high concentrations of genistein *in utero*, methylation of promotor regions of genes that code for estrogen metabolizing enzymes could thus be induced, consequently decreasing the transcription of *Comt* and *Nqo1* even later in life. As a result, the net effect of changes in gene expression presumably contributed to the generation of ROS and formation depurinating adducts.

The effects of *in utero* genistein exposure in offspring were strongest in mice that were heterozygous for the *Atm-*ΔSRI mutation. Male Atm-ΔSRI mice have impaired anti-oxidant capacity in their testes (19). Therefore, to investigate to what extent the antioxidant system was capable of preventing oxidative DNA damage and maintaining genetic integrity, the antioxidant status in mice with different *Atm-*ΔSRI genotypes and maternal diets was examined. The *Atm-*ΔSRI mutation resulted in significantly lower reduced GSH concentrations in testis of heterozygous mice, when compared to mice that did not carry the mutation. ATM regulates GSH regeneration from GSSG by glutathione reductase (GR), through enhancing G6PDH activity (Figure 1). G6PDH activity was significantly lower in *Atm-*ΔSRI heterozygous mice compared to wild-type mice. However, the maternal *in utero* genistein enriched diet significantly increased G6PDH activity in offspring, which might suggest a (partial) compensatory mechanism to counteract the increased formation of ROS.

In our study, a maternal genistein-rich diet in combination with a compromised antioxidant system, resulted in global hypermethylation, which may facilitate the transmission of the observed effects to adult life. However, it remains possible that increased levels of oxidative stress may affect DNA methylation, instead of DNA methylation affecting the processes that lead to oxidative stress. The design of the current study does not allow us to examine whether the changes in methylation already occurred earlier in life and preceded gene expression changes. Another limitation of the study is that estrogen metabolites were not measured, and therefore the hypothesis as depicted in Figure 1 remains speculative. Nevertheless, the current results support this hypothesis. The hypothesized model suggested that high genistein-intake during pregnancy may induce DNA damage in both the testes as well as germline cells. Consequently, the induction of oxidative DNA damage in testis and sperm of the F1 generation may also affect the genetic integrity of the F2 generation (33, 34). These effects may be notable especially in individuals with a compromised antioxidant system. Overall, since phyto-estrogens are increasingly integrated into our food chain, it is warranted to continue research on the induction of oxidative DNA damage in testes and germ cells of offspring by a genistein-rich maternal diet.

## Data availability statement

The raw data supporting the conclusions of this article will be made available by the authors, without undue reservation.

## Ethics statement

The animal study was reviewed and approved by Institutional Animal Care Committee of Maastricht University.

## Author contributions

RG and MJ wrote the manuscript. F-JS, SD-K, and KV were involved in the set up of the experiment and conducting the experiments. All authors approved the final version.

## Funding

This work was partly supported by grant number 06A031 from the American Institute for Cancer Research.

## Conflict of interest

The authors declare that the research was conducted in the absence of any commercial or financial relationships that could be construed as a potential conflict of interest. The handling editor MS declared a shared affiliation with the authors at the time of review.

## Publisher's note

All claims expressed in this article are solely those of the authors and do not necessarily represent those of their affiliated organizations, or those of the publisher, the editors and the reviewers. Any product that may be evaluated in this article, or claim that may be made by its manufacturer, is not guaranteed or endorsed by the publisher.
