# Supplementary material for: Maternal exposure to genistein during pregnancy and oxidative DNA damage in testes of male mouse offspring
Source: Front Nutr. 2022 Jul 18;9:904368. doi: 10.3389/fnut.2022.904368 (PMC9340160; doi:10.3389/fnut.2022.904368)
Supplement: Supplementary file 1 [file Table_1.docx]

# Supplementary information

**Supplementary table 1: Primers used in quantitative real-time PCR**

**Table 1A** Forward and reverse primers used in quantitative real-time PCR.

| **Gene** | **Forward primer (5'-3')** | **Reverse primer (5'-3')** |
| --- | --- | --- |
| *Cyp1b1* | AACGCAGCCGGTGATTGT | TGTACCGACAGCCGAAGCA |
| *Comt* | AGAGAAGGAGTGGGCCATGA | CCGAATCACTGCATCCATGA |
| *Nqo1* | CAGTTCCCATTGCAGTGGTTT | CCTGCTACGAGCACTCTCTCAA |

**Table 1B** Forward and reverse primers for different repetitive elements and endogenous reference, used in methylation-sensitive McrBC real-time PCR. IAP, intracisternal A-particle; LINE, long interspersed nucleotide element; SINE, short interspersed nucleotide element.

| **Repetitive element** | **Forward primer (5'-3')** | **Reverse primer (5'-3')** |
| --- | --- | --- |
| LINE | TTTGGGACACAATGAAAGCA | CTGCCGTCTACTCCTCTTGG |
| SINEB1 | GTGGCGCACGCCTTTAATC | GACAGGGTTTCTCTGTGTAG |
| SINEB2 | GAGATGGCTCAGTGGTTAAG | CTGTCTTCAGACACTCCAG |
| IAP-GAG | AGCAGGTGAAGCCACTG | CTTGCCACACTTAGAGC |
| major satellite | GACGACTTGAAAAATGACGAAATC | CATATTCCAGGTCCTTCAGTGTGC |
| minor satellite | CATGGAAAATGATAAAAACC | CATCTAATATGTTCTACAGTGTGG |
